# Understanding the Allosteric Modulation of PTH1R by a Negative Allosteric Modulator

**DOI:** 10.3390/cells12010041

**Published:** 2022-12-22

**Authors:** Mengrong Li, Yiqiong Bao, Ran Xu, Miaomiao Li, Lili Xi, Jingjing Guo

**Affiliations:** 1 College of Life Sciences, Nanjing Agricultural University, Nanjing 210095, China; 2 Office of Institution of Drug Clinical Trial, The First Hospital of Lanzhou University, Lanzhou 730000, China; 3 Centre in Artificial Intelligence Driven Drug Discovery, Faculty of Applied Science, Macao Polytechnic University, Macao 999078, China; 4 Engineering Research Centre of Applied Technology on Machine Translation and Artificial Intelligence, Macao Polytechnic University, Macao 999078, China

**Keywords:** class B GPCR, parathyroid hormone type 1 receptor, negative allosteric modulator, molecular dynamics simulation, allosteric regulation

## Abstract

The parathyroid hormone type 1 receptor (PTH1R) acts as a canonical class B G protein-coupled receptor, regulating crucial functions including calcium homeostasis and bone formation. The identification and development of PTH1R non-peptide allosteric modulators have obtained widespread attention. It has been found that a negative allosteric modulator (NAM) could inhibit the activation of PTH1R, but the implied mechanism remains unclear. Herein, extensive molecular dynamics simulations together with multiple analytical approaches are utilized to unravel the mechanism of PTH1R allosteric inhibition. The results suggest that the binding of NAM destabilizes the structure of the PTH1R–PTH–spep/qpep (the C terminus of Gs/Gq proteins) complexes. Moreover, the presence of NAM weakens the binding of PTH/peps (spep and qpep) and PTH1R. The intra- and inter-molecular couplings are also weakened in PTH1R upon NAM binding. Interestingly, compared with our previous study of the positive allosteric effects induced by extracellular Ca^2+^, the enhanced correlation between the PTH and G-protein binding sites is significantly reduced by the replacement of this negative allosteric regulator. Our findings might contribute to the development of new therapeutic agents for diseases caused by the abnormal activation of PTH1R.

## 1. Introduction

G protein-coupled receptors (GPCRs) act as environmental sensors that are activated by a variety of extracellular activating ligands (for example, hormones, ions, neurotransmitters, peptides, and lipids) and conduct external environmental signals from the extracellular matrix to the cytoplasm [1,2]. It has been demonstrated that multiple diseases are associated with GPCRs, such as Alzheimer’s disease, cancer, diabetes, depression, and cardiovascular disease, accounting for approximately one-third of all marketed drugs targeting GPCRs [2,3]. Upon binding to agonists, GPCRs interact with intracellular partners to induce different cytoplasmic signaling pathways [4].

The parathyroid hormone (PTH) type 1 receptor (PTH1R) is a typical member of the class B GPCR with important functions involved in the regulation of the skeletal, cardiovascular, and endocrine systems [5,6]. The activation of PTH1R by the agonist PTH and PTH-related peptide (PTHrP) triggers the Gs/cAMP and Gq/Ca^2+^ signaling pathways [7]. The distinction between PTH and PTHrP is that the cAMP response differs markedly in its duration and cellular localization [8,9]. The internalization of the PTH-PTH1R complex promotes sustained cAMP production [8], while the activation of the receptor by PTHrP produces only transient cAMP generation at the cell membrane [9].

With technological breakthroughs, cryo-electron microscopy structures are essential to understanding receptor activation and downstream signaling cascades [10,11,12,13,14]. PTH1R contains an N-terminal extracellular domain (ECD) and a transmembrane domain (TMD) [15]. Through the recognition and anchoring of orthosteric ligands by ECD and TMD, the outward movement of the cytoplasmic end of transmembrane helix 6 (TM6), forming a sharp kink, together with the slight inward movement of transmembrane helices 5 and 7 (TM5 and TM7), suggests a co-activation mechanism of class B GPCR [10,16]. Hence, the activation of GPCR is an allosteric process that couples agonist binding with G-protein recruitment. Besides ligands binding at the orthosteric sites, many allosteric effectors occupying distal sites could also cause conformational and/or dynamics changes to GPCR, leading to cascade reactions. These GPCR allosteric modulators further increase the complexity of GPCR functioning [17]. The complex GPCR allosteric ligands could be categorized as: (i) positive allosteric modulators (PAM) that enhance the GPCR response; (ii) negative allosteric modulators (NAM) that decay the receptor response; or (iii) neutral modulators that do not affect the activity of the orthosteric ligands [18]. Moreover, PAMs and NAMs are able to potentiate/diminish downstream signaling by differentially affecting the binding affinity and efficacy of orthosteric ligands [19,20].

As the essential target for the treatment of bone and mineral ion disorders, the activation and allostery of PTH1R have attracted great interest [19]. On the one hand, experimental and computational methods have elucidated that extracellular Ca^2+^ could act as a positive allosteric modulator of PTH1R by prolonging the receptor activation and enhancing endosomal cAMP signaling [20,21,22]. Our previous works illuminated that stable local Ca^2+^-coupling structures induced enhanced allosteric signaling between PTH1R and G protein, and these results contributed to the treatment of calcium homeostasis-related diseases [21,22]. On the other hand, PTH1R overexpression caused symptoms of tissue hypoplasia, and the inhibition of overexpression might play a key role in the treatment of diseases [19]. A more recent study indicated that Pitt12 (MolPort ID: 039-313-655), binding at a druggable site located within the PTH1R, caused a decrease in the kinetics and magnitude of G proteins (Gs and Gq) association with the PTH1R, serving as an allosteric antagonist candidate for the treatment of hyperparathyroidism [23]. However, the implied molecular mechanism of this kind of allosteric inhibition remains unclear.

Molecular dynamics (MD) simulation is a powerful way to characterize the structure–function relationship for both membranes and proteins [24,25,26], and there are many excellent computational studies for GPCR-G protein systems [27,28]. Here, we explored the allosteric effects of the negative allosteric modulator Pitt12 (hereafter referred to as NAM) on the binding between PTH1R and the C terminus of Gs and Gq proteins (hereafter referred to as spep and qpep) via extensive classical MD simulations, as well as enhanced sampling-steered MD simulations. First of all, the overall stability of PTH1R in the presence of NAM was investigated by RMSD and RMSF analyses. Next, our results suggested that NAM affected the binding between either PTH or peps (spep and qpep) and PTH1R, which might potentially explain the allosteric inhibition of PTH1R activity upon NAM binding. Moreover, it was revealed that NAM might reduce the activation of PTH1R allosterically by affecting conformational dynamics. Hence, an in-depth understanding of PTH1R allosteric inhibition will help establish the fundamentals of PTH1R allosteric signaling and the associated allosteric drug design.

## 2. Methods

### 2.1. Structure Preparation

**Figure 1:**
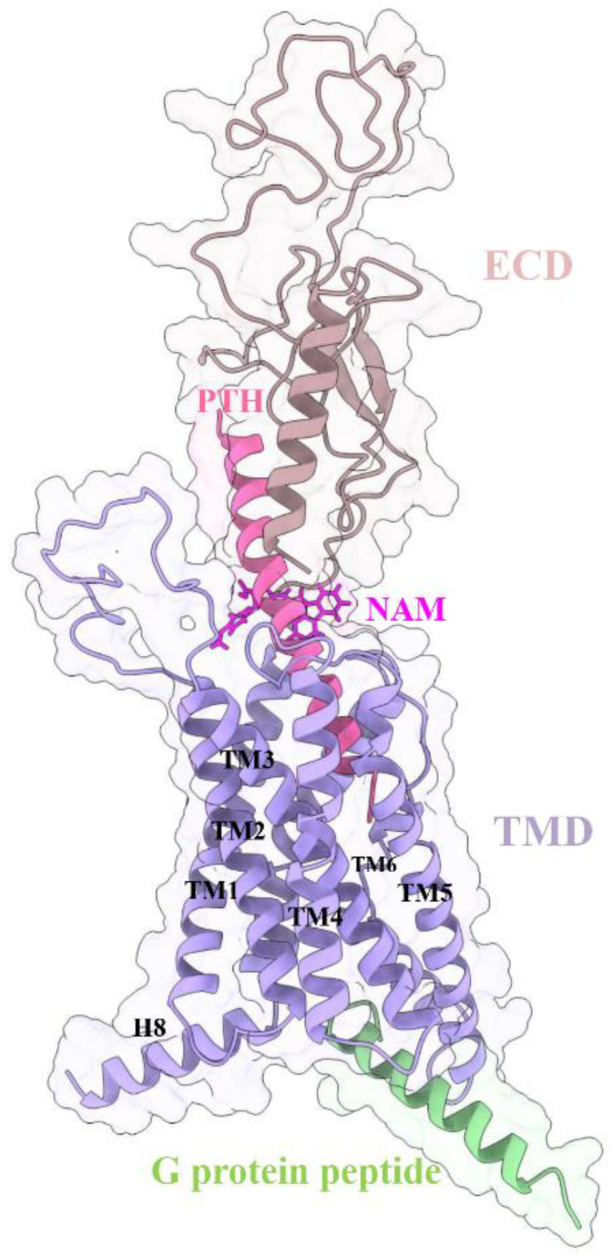
The system information of the modeled PTH1R complex. The protein and peptides are shown as cartoons, and the NAM molecule is shown as sticks colored in magenta. ECD, brown; TMD, purple; PTH, hotpink; G protein peptide, green.

We used the 3.0-Å cryo-EM structure of long-acting PTH (LA-PTH)-PTH1R-Gs complex (PDB ID: 6nbf [10]) as the initial PTH1R model, and the LA-PTH analog was replaced by agonist PTH (PDB ID: 1et1 [29]). The Modeller [30] plugin in Chimera [31] was used to model the absent structures of PTH1R (ECD residues 56−104, ECL1 residues 247−275, and intracellular loop 3 (ICL3) residues 394−398). The structures of Nb35, palmitic acid, and cholesterol were removed, while the C terminus of Gs protein (spep, residues 368−394) was reserved. The qpep (residues 333−359) was homology modeled from sequence alignment to the spep. Thus, we obtained the PTH1R–PTH–spep (Figure 1) and PTH1R–PTH–qpep complexes. Then, two complexes were taken as the docking receptor structures. The structures of receptor and ligand (MolPort ID: 039-313-655) were processed using Schrödinger as in previous studies [23,32]. The ligand was prepared using LigPrep module with an OPLS-2005 force field and docked to the allosteric site of PTH1R using the standard precision (SP) mode. Then, these complexes were inserted into a pre-equilibrated POPC lipid bilayer in CHARMM-GUI Builder [33]. The systems were solvated with TIP3P [34] water and neutralized by adding counter ions to a concentration of 150 mM NaCl. Finally, four systems were obtained, including PTH1R–PTH–spep, PTH1R–PTH–spep–NAM, PTH1R–PTH–qpep, and PTH1R–PTH–qpep–NAM.

### 2.2. Molecular Dynamics Simulation

For all systems, molecular dynamics simulations were carried out using the AMBER 18 package [35]. The complexes were described by the AMBER14SB [36], GAFF [37], and Lipid14 [38] force fields. The distance between the solute and the edge of the solvent box was set to 10 Å [39]. Additionally, the SHAKE [40] algorithm was used to constrain bond vibrations involving hydrogen. Three independent parallel molecular dynamics simulations were performed for each system. The MD simulations involved four steps: energy minimization, heating, equilibration, and production protocols. The first four 25,000-step minimizations were performed using the steepest descent and the conjugate gradient algorithm. Subsequently, all systems were heated from 0 to 310 K at 50 ps. Next, each system was equilibrated in the NVT and NPT ensembles with force constants of 1.0 and 0.5 kcal/mol/Å^2^ applied to non-hydrogen atoms and heavy atoms of proteins for 10 and 50 ns, respectively. Finally, the 1000 ns production run was simulated without any restraint at the NPT ensemble. Coordinates of the production runs were recorded every 5 ps and MD analyses were carried out using the CPPTRAJ module [41].

### 2.3. Steered Molecular Dynamics (SMD) Simulation

In our work, the SMD simulations were performed using the AMBER 18 package [35]. A force was applied to the PTH spiral mass of center (COM), and PTH was pulled out slowly from the binding pocket for 20 Å. Three independent 5-ns SMD simulations were performed with a spring constant of 10 kcal/mol/Å^2^.

### 2.4. Principle Component Analysis

A principal component analysis was carried out using the CPPTRAJ module [41] in AMBER 18 [35] over a total of 360,000 conformations (90,000 frames evenly sampled from each simulation). For each system, the results were converted into a free energy surface over the first two principal components (PC1 and PC2) according to the Boltzmann relation [42].

### 2.5. Binding Free Energy Calculation

To obtain more statistically significant results, the binding free energy between PTH1R and PTH was calculated for the last 300 ns using the MM/GBSA approach [43]. To apply the method, we extracted the multiple snapshots without water molecules, counter ions, and membranes from the MD trajectories. For each system, the free energy was calculated for each molecular species (complex, receptor, and ligand), and the binding free energy was computed as below [44]:

Δ*G*_bind_ = Δ*G*_complex_ − Δ*G*_receptor_ − Δ*G*_ligand_

### 2.6. Dynamical Network Analysis

The community network analysis of each system was performed using the NetworkView [45] plugin in VMD [46] for the last 300 ns MD trajectories. A protein network was created, with residues and their contacts acting as nodes and edges. An edge would be created if heavy atoms between two nodes except adjacent ones were within a 4.5 Å cutoff distance for at least 75% of the MD trajectory. Then, the correlation data between residues was used to weigh the edges in the dynamical network. Sequences with adjacent residues were excluded and could not be joined by an edge. Next, the original network was partitioned into subnetworks, named communities here. Nodes within a community had more and stronger connections than nodes in other communities. The inter-community cumulative betweennesses, calculated based on the edge lying between communities, was a measure of the strength of the inside coupling of the system.

### 2.7. Anisotropic Network Model (ANM)

The Anisotropic Network Model web server [47] (ANM 2.0) with the default setting was used to explore the coupled movements of the protein, including allosteric changes. We performed the ANM approach to computing the conformational mobility profiles in the essential space of low-frequency modes. Based on the representative conformations during the last 300 ns of each system, 20 normal modes were finally obtained.

## 3. Results and Discussion

### 3.1. PTH1R Complex Binding with NAM Favors the Flexible Conformation

After MD simulation, NAM was bound stable and close to the stem region (connecting ECD and transmembrane helix 1), as was shown in a recent study [23]. There was a hydrogen bond between NAM and PTH1R, and most of the contact residues were polar and hydrophobic residues (Figure S1), indicating the importance of polar and hydrophobic interactions. Firstly, the root-mean-square deviations (RMSDs) of Cα atoms of the seven-transmembrane (7TM) helices were monitored. As Figure S2 showed, RMSDs of each system were stable during the MD simulations with most values smaller than 3 Å, especially during the last 300 ns. Therefore, most of the further analyses were based on the equilibrated trajectories.

**Figure 2:**
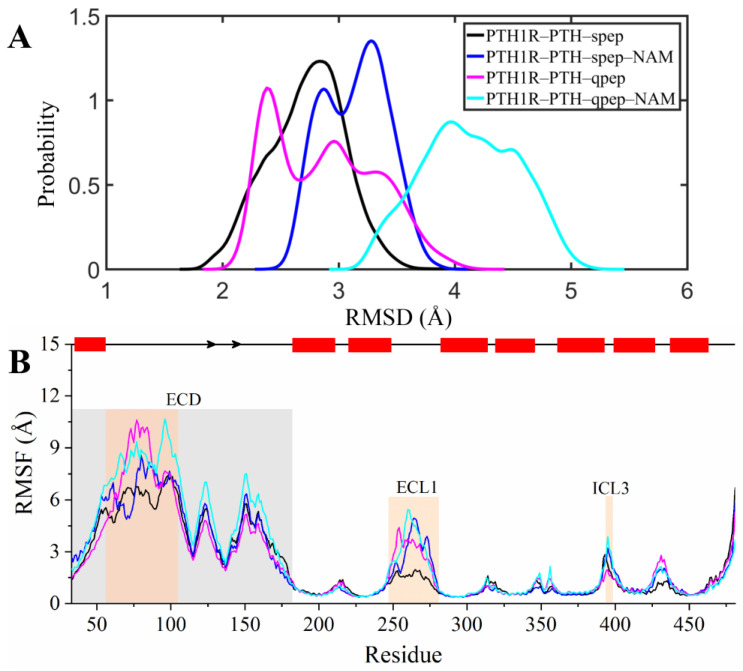
The Cα RMSD probability distribution profiles (**A**) and RMSF (**B**) values during the last 300 ns. The rectangles and arrows on the top represent ordered secondary structures for α-helices and β-sheets, respectively. The ECD is highlighted by shading in gray, and modeled regions in ECD, ECL1, and ICL3 are highlighted in orange.

Based on the distribution of the 7TM Cα RMSD values considering all three replicates (Figure 2A), we found that the presence of NAM shifted the system to more flexible conformational states with larger RMSDs, indicating the high stability of non-NAM binding systems during the simulations. Then, in order to investigate the effects of NAM binding on PTH/peps binding and the dynamic behaviors of PTH1R, root-mean-square fluctuations (RMSFs) were calculated based on the last 300 ns MD trajectories. All systems presented similar RMSF trends (Figure 2B, Figures S3 and S4). Both the N-terminal of PTH inserted into the receptor and the C-terminal of peps exhibited quite low flexibility owing to a powerful association with the receptor, indicating the high stability of these regions’ binding with receptors (Figure S3). Moreover, as illustrated in Figure 2B, it was noted that the RMSF values of the ordered secondary structures were smaller than 2.0 Å, suggesting the overall stability of the receptor. In the NAM-bound systems, the overall protein skeleton fluctuation was relatively higher, especially in the disordered loops. We also observed that the ECD of PTH1R was more flexible than the TMD in all systems, which is consistent with the flexible ECD structural characteristics [10].

As can be seen in Figure 2B, the high flexibility of ECL1 was reduced in non-NAM-bound systems compared to NAM-bound systems, especially the PTH1R–PTH–spep system. Previous studies demonstrated the critical role of PTH1R in the regulation of Ca^2+^ homeostasis and bone turnover [19]. In addition, extracellular Ca^2+^ could serve as a PAM to increase PTH1R activation signaling, and ECL1 acts as a binding requisite of extracellular calcium ions [20,21,22]. Thus, the structural flexibility of PTH1R ECL1 influenced by the binding of NAM might also provoke allosteric signal propagation from the extracellular to the intracellular matrix.

In addition, different ECD and ECL1 conformations in class B1 GPCR may meet different activation requirements [48,49]. In the glucagon subfamily (for instance, glucagon-like peptide-1 receptor [50] and growth hormone releasing hormone receptor [14]) and the PACAP/VIP subfamily (such as pituitary adenylate cyclase activating polypeptide 1 receptor [51]), ECL1 extends around the endogenous ligand to form a broad interaction. Therefore, the C-terminus of the endogenous ligand is recognized by both ECD and ECL1. Due to its conformational variability, no structural features of ECL1 have been resolved experimentally in the PTH subfamily (including PTH1R and parathyroid hormone receptor 2) [10,52]. The flexible ECD and ECL show their ability to regulate and diversity in regulating different class B1 receptors. Indeed, more high-resolution structures remain to be acquired in order to support additional possibilities in the future.

### 3.2. NAM Binding Alters the Conformational Dynamics of PTH1R TMD Region

**Figure 3:**
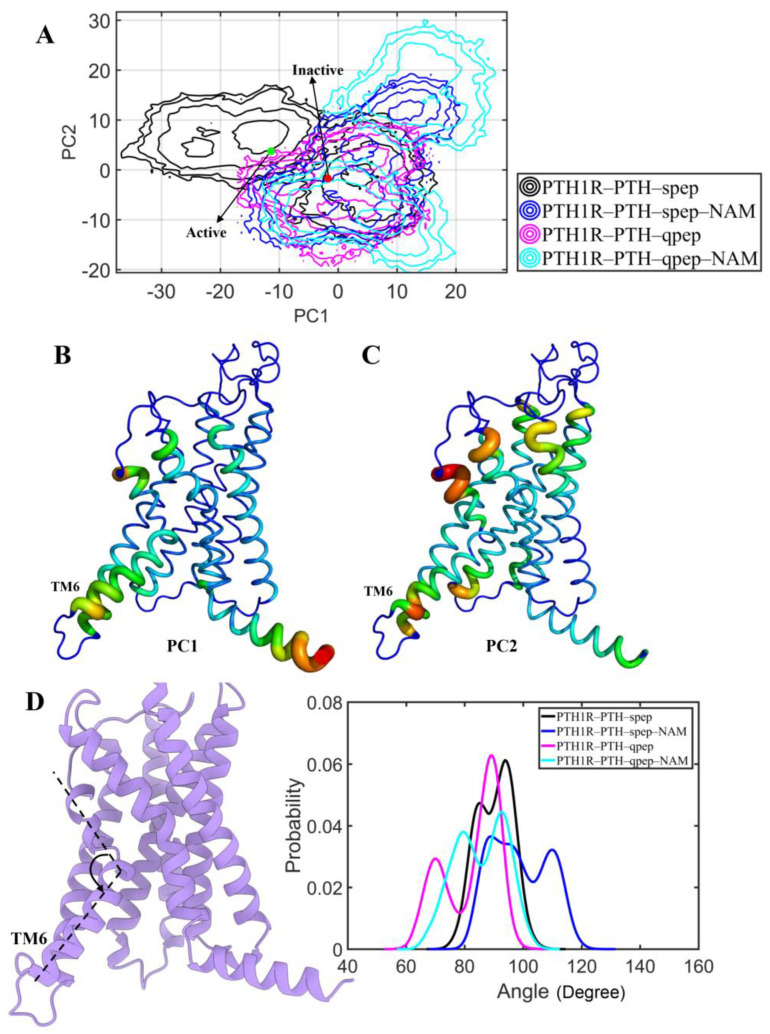
Conformational changes of PTH1R TMD in all systems. (**A**) The first two principal components (PC1 and PC2) are shown in free energy surfaces. PC coordinates of active and inactive crystal structures are shown as green and red dots. (**B**,**C**) Sausage presentation with the width and color (from blue to red) related to the conformational differences represented by PC1 and PC2. (**D**) The kink angle at the middle of TM6 for each system.

To further characterize the conformational differences among the four systems, a principal component analysis was carried out for the last 300 ns MD trajectories. The corresponding results were shown in the free energy surface using the first two principal components, PC1 and PC2 (Figure 3). It was noted that the energy basins of all systems were close to each other. The conformational sampling of the PTH1R–PTH–qpep–NAM system had two local minima, while other systems were relatively concentrated. Moreover, the NAM-bound form was separated from the related non-NAM-bound one, mainly along PC1 or PC2. As shown in Figure 3B and Movie S1, PC1 mainly captured the closing motion of the intracellular G protein binding region, which was associated with the inhibition of PTH1R activation [10]. Additionally, PC2 illustrated the inward motion of the PTH binding site and intracellular G protein binding region (Figure 3C and Movie S2). Additionally, the locations of active and inactive crystal structures [10,53] were consistent with the movement of PC1 (Figure 3).

Moreover, the activation of GPCR was mainly accompanied by a sharp kink in the middle of the TM6 [54]. The kink angle at the middle of TM6 was further calculated for each system (Figure 3D) to explore the effects of NAM. Compared with the corresponding non-NAM-bound system, the presence of NAM resulted in a smaller curvature of TM6 with larger kink angles. Therefore, the binding of NAM might promote the dynamics of PTH1R (in line with the RMSD and RMSF data) and the inactive conformation.

### 3.3. The Presence of NAM Destabilizes the Binding between PTH/Peps and PTH1R

It was verified that the binding of NAM reduced the stability of the receptor and induced dynamical and conformational perturbations. To further confirm the negative allosteric effects of NAM on the activation of the receptor, the binding affinity of PTH and peps on the last 300 ns MD trajectories of each system was calculated. As for the PTH, the MM/GBSA method was used to measure the binding affinity between PTH and PTH1R directly, and detailed contributions of various energy components were presented in Table 1. It can be seen that the combination of PTH1R and PTH was diminished in both NAM-bound systems. The binding free energy for each system had a broad distribution, which was mainly due to the polar interactions including direct electrostatic and polar desolvation interactions. Without the NAM, the binding of PTH in the spep system was stronger than that in the qpep system, with a difference of about 9 kcal/mol. After NAM binding, the affinity between PTH and the receptor in the spep system was reduced more than that in the qpep system, and the two NAM-bound systems were close to each other. These results were consistent with a recent study based on single-cell fluorescence resonance energy transfer (FRET) assays, which confirmed that NAM induced the diminished binding of G proteins to the receptor [23].

**Figure 4:**
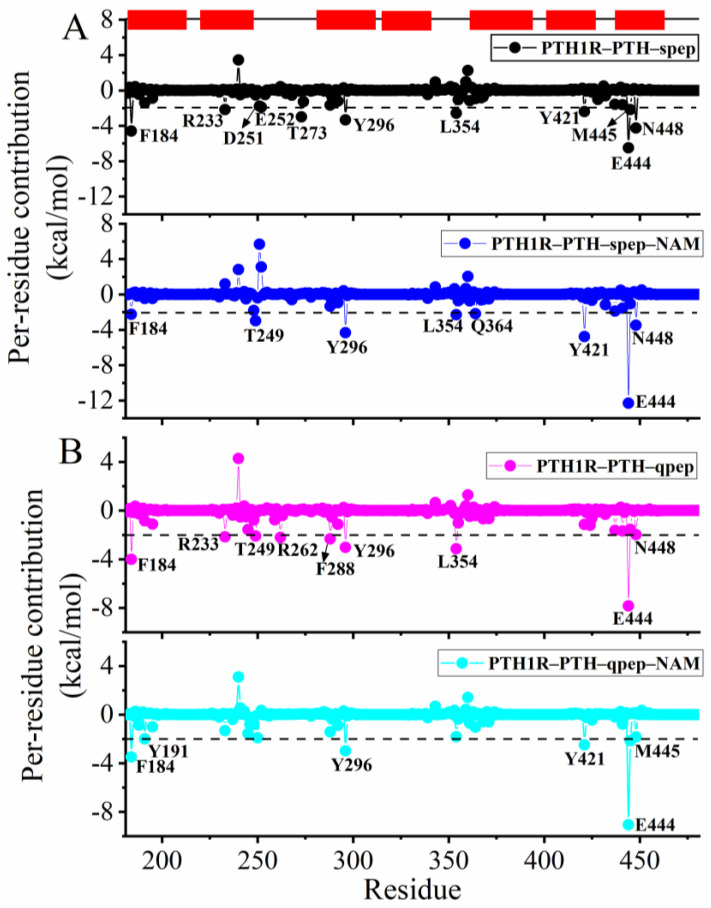
Residue contribution to the binding of PTH: (**A**) PTH1R–PTH–spep and PTH1R–PTH–spep–NAM systems; (**B**) PTH1R–PTH–qpep and PTH1R–PTH–qpep–NAM systems.

Next, the contribution of each residue to the binding of PTH was investigated to further identify key residues affected by NAM binding. As Figure 4 shows, most of the identified hot spot residues for PTH1R-PTH binding were located in transmembrane helices 1/2/3/5/6/7 and ECL2, including F184, Y191, R233, F288, Y296, L354, Q364, Y421, E444, M445, and N448, which was consistent with previous experimental results [10]. Upon the binding of NAM, the contributions of many residues were lower than those in the non-NAM bound systems.

**Figure 5:**
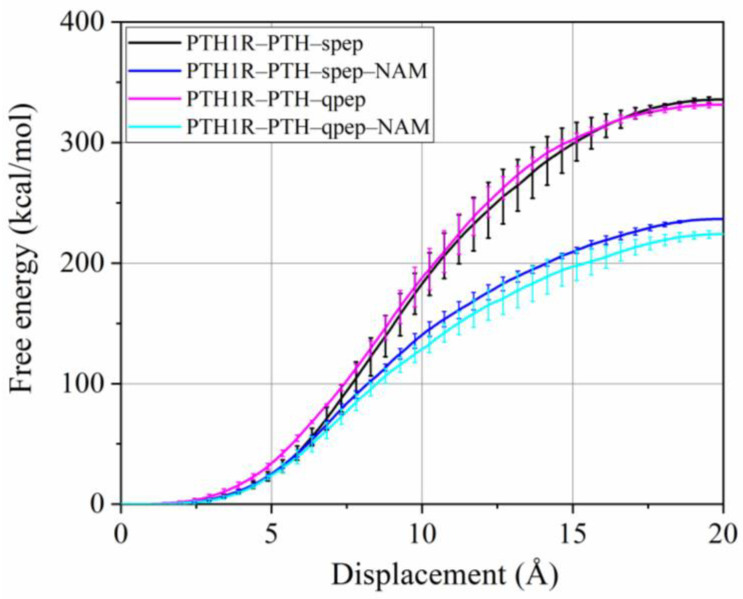
Free energy profiles with error bars from SMD simulations for PTH dissociation of each system.

Moreover, Steered molecular dynamics (SMD) simulation is a technique that allows for the manipulation of a system from an initial configuration to a target configuration by applying an external force [55]. Next, three replicates of SMD simulations were performed to further investigate the effects of NAM on the binding or dissociation of PTH (Figure 5). In keeping with the above results, the non-NAM-bound systems had stronger external work than the other two, and the results for the two peps systems were similar.

**Figure 6:**
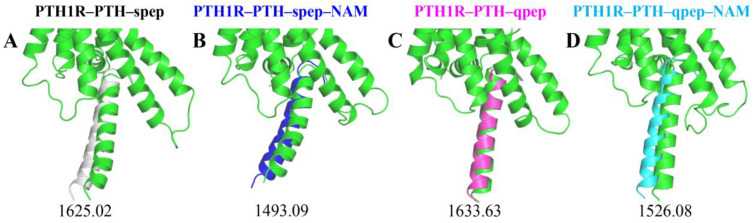
Cartoon illustration of the predicted spep/qpep position of each system in ZDOCK. (**A**) PTH1R–PTH–spep system; (**B**) PTH1R–PTH–spep–NAM system; (**C**) PTH1R–PTH–qpep system; and (**D**) PTH1R–PTH–qpep–NAM system. The representative conformation of the predominant cluster during the last 300 ns in each system is shown in green. The numbers below correspond to the ZDOCK binding score.

On the other hand, we estimated the binding of peps by redocking them to the representative conformation of PTH1R in each system utilizing the ZDOCK server [56]. As illustrated in Figure 6, the best binding pose of each system was very similar to the original conformation, indicating that the docking results were reasonable. The docking scores for PTH1R–PTH–spep, PTH1R–PTH–spep–NAM, PTH1R–PTH–qpep, and PTH1R–PTH–qpep–NAM systems were 1625.02, 1493.09, 1633.63, and 1526.08, respectively. These results suggested that after conformational rearrangements under different conditions, the non-NAM-bound systems had stronger binding capacities than the NAM-bound ones.

Therefore, it can be concluded based on the MM/GBSA, steered MD simulations, and ZDOCK docking results that the presence of NAM allosterically inhibits the interaction between PTH1R and PTH and possibly suppresses the response of the receptor to peps. Thus, it was demonstrated that NAM had allosteric inhibition effects on the binding of PTH1R and PTH, which might subsequently affect the activation of the receptor and the binding of G proteins.

### 3.4. Observation of Weakened Intra- and/or Inter-Molecular Coupling upon NAM Binding

**Figure 7:**
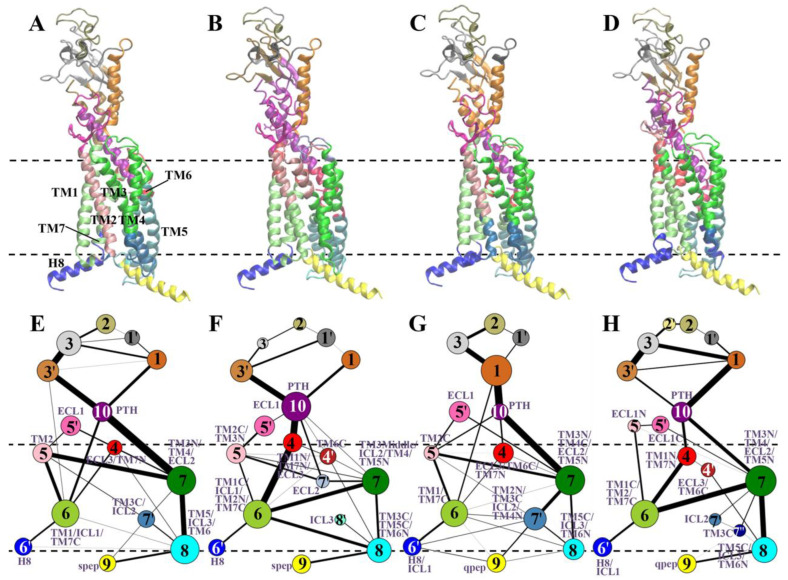
Community network for each system. Communities are highlighted as circles with different colors, which are consistent across all systems. The inter-community cumulative betweennesses are displayed by the thickness of lines connecting communities. (**A**,**E**) PTH1R–PTH–spep system; (**B**,**F**) PTH1R–PTH–spep–NAM system; (**C**,**G**) PTH1R–PTH–qpep system; and (**D**,**H**) PTH1R–PTH–qpep–NAM system.

Protein dynamic networks have been widely applied to explain heterogeneous mechanisms [21,32,57]. Here, we employed this method to explore the effects of ligand/peps binding on the residue–residue communication of PTH1R. Protein dynamic networks can be represented using communities (subnetworks), where the number of members of each community is indicated by the size of the nodes, and the shared information between communities is shown by the thickness of the edges. Although the NAM-bound system exhibited a larger number of communities than other systems, the community maps for all systems had similar frames (Figure 7). As illustrated in Figure 7A,E, they can be considered several common parts: (1) the ECD of PTH1R (communities 1–3); (2) the TMD of PTH1R (communities 4–8); (3) the peps (community 9); and (4) the PTH (community 10). The number of communities within the ECD of PTH1R was the same in both spep systems, but the communication within ECD became weaker upon the binding of NAM. There were more small communities in the PTH1R–PTH–qpep–NAM system than in the PTH1R–PTH–qpep system, indicating that the original residue–residue couplings had been impaired. Hence, the presence of NAM might reduce the stability of ECD. These findings were consistent with the RMSF results of PTH1R ECD (Figure 2B).

PTH and NAM belonged to community 10, and the residue–residue couplings within the PTH and transmembrane helices were reduced by the enhanced number of communities (communities 4, 5, and 7) and/or the decreased inter-community cumulative betweennesses in the NAM-bound system (Figure 7F,H). Furthermore, in the intracellular G protein binding sites including communities 6–8, it was demonstrated that the number or the size of these communities, especially communities 7 and 8, was increased in NAM-bound systems (Figure 7F,H).

### 3.5. The Binding of NAM Altered the Routes for Allosteric Signal Propagating from Extracellular to Intracellular

**Figure 8:**
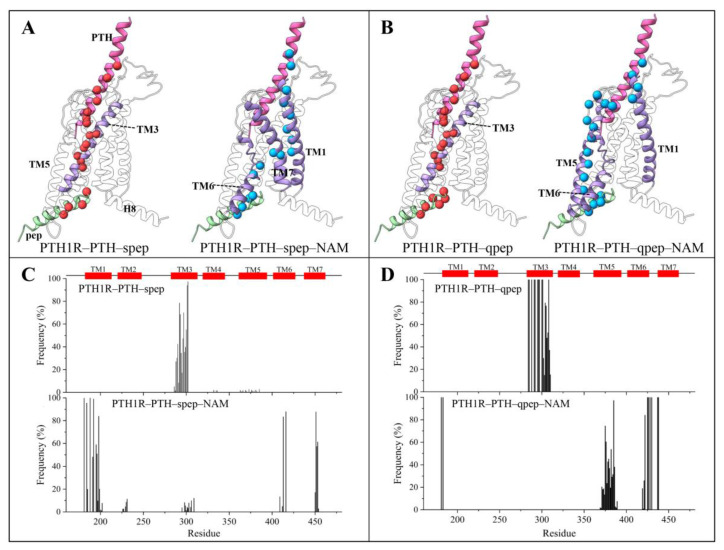
Allosteric signal propagating paths from the PTH to peps. (**A**,**B**) Optimal paths of each system are colored red and blue, respectively. (**C**,**D**) The frequencies of residues involved in all recognized pathways are displayed on the structures.

To further explore the molecular basis of the allosteric effects induced by NAM, potential allosteric pathways were identified between PTH in the orthosteric site and peps in the cytoplasmic site. We calculated suboptimal paths from PTH E22 (interacts with the NAM and related to the Ca^2+^ allostery induced the G proteins cascade signals [20,21]) to peps based on a dynamical protein network for the NAM-induced allosteric signals. Suboptimal paths were generated from the aforementioned dynamical network matrix. The key residues of the optimal allosteric pathways in the absence and presence of NAM were shown as red and blue spheres, respectively (Figure 8A,B). In the PTH1R–PTH–spep and PTH1R–PTH–qpep systems, the key residues were located in similar regions (TM3) in the receptor TMD. However, in the NAM-bound systems, the signals propagated via longer paths, from the PTH via TM1-6-7 or TM1-6-5 of PTH1R and eventually to the peps (Figure 8A,B), which was consistent with the aforementioned community network results (Figure 7). Moreover, the frequency of residues involved in all recognized pathways was calculated (Figure 8C,D). The locations of high-frequency residues were well in line with those in the shortest optimal path. Hence, a lower cooperative allosteric network upon NAM binding was further confirmed.

As an intrinsically allosteric protein [58], the activation of PTH1R is induced by an agonist and transmitted to the intracellular binding site. With a decrease in residue–residue coupling within the PTH-TMD and the increasing number of communities on the intracellular side of TMD after NAM binding, the binding of PTH1R to peps appears to be allosteric diminished. Therefore, in the presence of NAM, the decreased allosteric signaling could transmit from the PTH binding pockets to the intracellular peps binding sites.

### 3.6. NAM Binding Induces Propagation of the Negative Allosteric Signal

**Figure 9:**
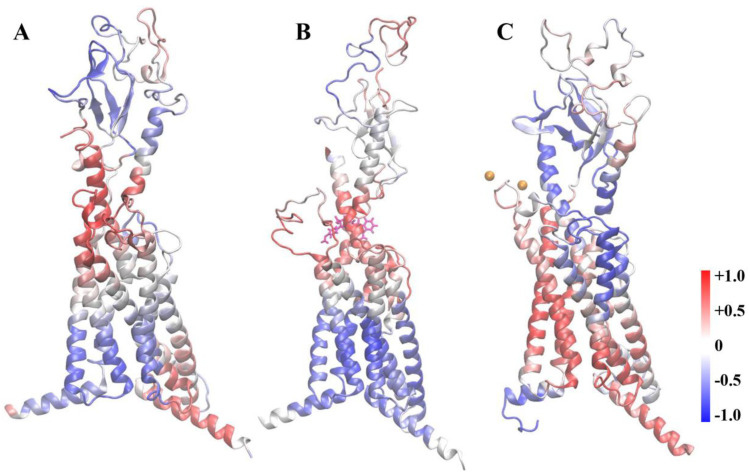
ANM results of the PTH1R–PTH–spep system (**A**), PTH1R–PTH–spep–NAM system (**B**), and PTH1R–PTH–spep–Ca^2+^ system (**C**). The ribbon representation of the complex is colored by the correlation strength of all residues with PTH E22 that coordinate with NAM (correlated, red; anticorrelated, blue). NAM molecule is shown as sticks colored in magenta, and Ca^2+^ ions are shown as orange spheres.

The anisotropic network model (ANM) acts as one of the computational methods to obtain protein allosteric network signaling from MD simulations [47]. The lowest-frequency ANM modes, also referred to as global modes, could represent the dynamic intrinsic conformational changes of proteins including allosteric changes [22,59]. Here, we performed ANM analyses to explore the allosteric effects of NAM on PTH1R (Figure 9). The cross-correlation values between PTH E22 and all other residues were mapped to the structure. Correlation residues were colored in red, anticorrelated in blue, and uncorrelated in white. In the PTH1R–PTH–spep system (Figure 9A), a positive correlation was observed between the PTH binding site and the G protein binding site. However, an inverse correlation between the two sites suggested a negative allosteric regulation of PTH1R by NAM (Figure 9B), which was consistent with results discussed earlier. In contrast, our previous work [22] found an enhanced correlation from the PTH binding site to spep via TMD in the presence of Ca^2+^, a positive allosteric regulator of PTH1R (Figure 9C). The comparison of the ANM results for the three systems suggested that the intracellular regions contributing to G-protein binding were allosterically coupled to the PTH-binding site, which might be affected by extracellular modulators, such as small molecules or ions. Overall, the protein dynamic networks, suboptimal path, and ANM results validated that NAM might provoke the propagation of the negative allosteric signaling of PTH1R.

## 4. Conclusions

This study used extensive MD simulations to characterize how a NAM impacted the conformations and dynamics of the PTH1R–PTH–peps complex. In particular, it was demonstrated that the existence of NAM allosterically diminished the PTH1R and PTH/peps association, which might further inhibit the activation of the receptor. Our results also suggested that the conformational dynamics of the system were increased upon the binding of NAM, resulting in a more flexible state. Moreover, using the dynamical network analysis, we deciphered how the binding of NAM could reduce the activation of PTH1R and weaken both intra- and inter-PTH1R couplings. In addition, our findings elucidated the allosteric signaling from the NAM coupling site to the intracellular G protein binding site and the negatively correlated motility of the two binding sites by the suboptimal path and ANM results. In summary, our simulations revealed the possible allosteric mechanisms involved in PTH1R inhibition and could benefit our understanding of the activation of PTH1R. Our present work is a qualitative determination of the negative feedback action of NAM on G-protein recruitment that does not discriminate based on mediated Gs/Gq signaling, which requires further experimental validation. Furthermore, in the future, more allosteric drugs targeting PTH1R should be designed and investigated to combat various diseases.

## Figures and Tables

**Table 1:** The components of the PTH binding free energy (kcal/mol) of each system.

	System	spep	spep + NAM	qpep	qpep + NAM
Contribution					
Δ*E*_vdw_		−200.10 ± 21.16	−218.30 ± 10.97	−192.93 ± 17.69	−186.14 ± 15.36
Δ*E*_ele_		−356.22 ± 50.99	−317.73 ± 60.58	−377.35 ± 108.46	−425.62 ± 105.72
Δ*G*_pol,sol_		455.96 ± 47.06	451.76 ± 52.62	478.47 ± 103.87	521.09 ± 108.65
Δ*G*_npol,sol_		−29.58 ± 2.05	−32.31 ± 1.53	−28.49 ± 3.28	−27.89 ± 2.55
Δ*E*_MM_		−556.32 ± 53.07	−536.03 ± 58.59	−570.28 ± 120.68	−611.76 ± 114.93
Δ*G*_sol_		426.38 ± 46.59	419.45 ± 52.69	449.98 ± 101.18	493.20 ± 106.62
**Δ*G*_MM/GBSA_**		−129.94 ± 17.40	−116.58 ± 13.21	−120.30 ± 15.24	−118.56 ± 15.19

## Data Availability

The data presented in this study are available on request from the corresponding author.

## Supplementary Materials

The following supporting information can be downloaded at: https://www.mdpi.com/article/10.3390/cells12010041/s1. Figure S1: Interaction between PTH1R and NAM after 1000 ns MD simulations. P: chain of PTH; R: chain of PTH1R. Figure S2: RMSDs of 7TM Cα atoms for all MD simulations. (A) PTH1R–PTH–spep system; (B) PTH1R–PTH–spep–NAM system; (C) PTH1R–PTH–qpep system; (D) PTH1R–PTH–qpep–NAM system. Figure S3: RMSF of peps (A) and PTH (B) in the last 300 ns MD trajectories using their average structures as references. Figure S4: RMSFs of each system in the last 300 ns MD trajectories using average structure as references: (A) peps; (B) PTH; (C) PTH1R. The rectangles and arrows on the top represent ordered secondary structures α-helices and β-sheets, respectively. Movies S1 and S2: The motions of PTH1R TMD along PC1 (S1) and PC2 (S2).
