# Supplementary material for: Understanding the Allosteric Modulation of PTH1R by a Negative Allosteric Modulator
Source: Cells. 2022 Dec 22;12(1):41. doi: 10.3390/cells12010041 (PMC9818451; doi:10.3390/cells12010041)
Supplement: Supplementary file 1 [file cells-12-00041-s001.zip › cells-2068692-supplementary.pdf]

Supplementary Materials

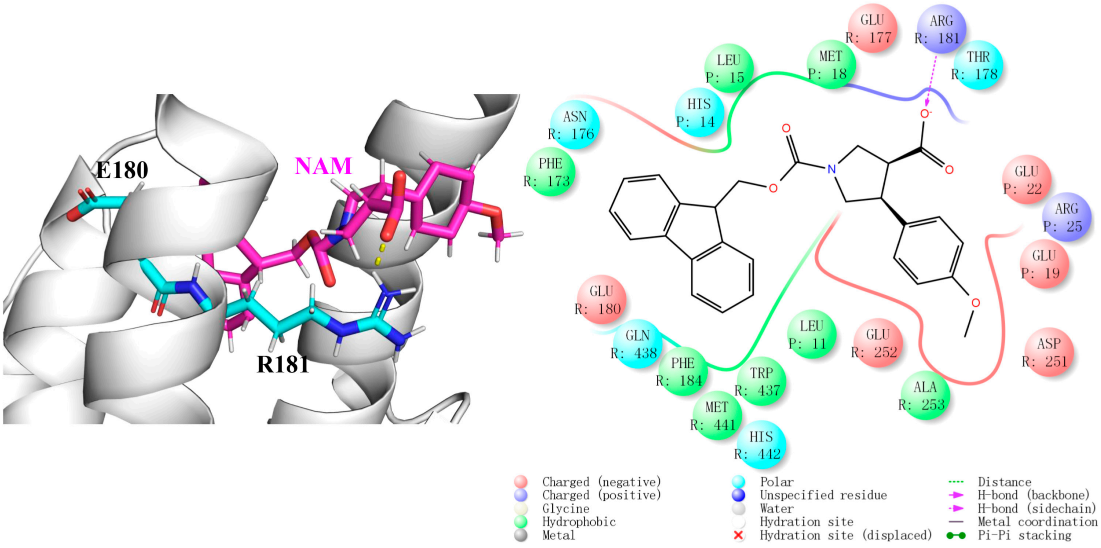

Figure S1. Interaction between PTH1R and NAM after 1000-ns MD simulations. P: chain of PTH; R: chain of PTH1R.

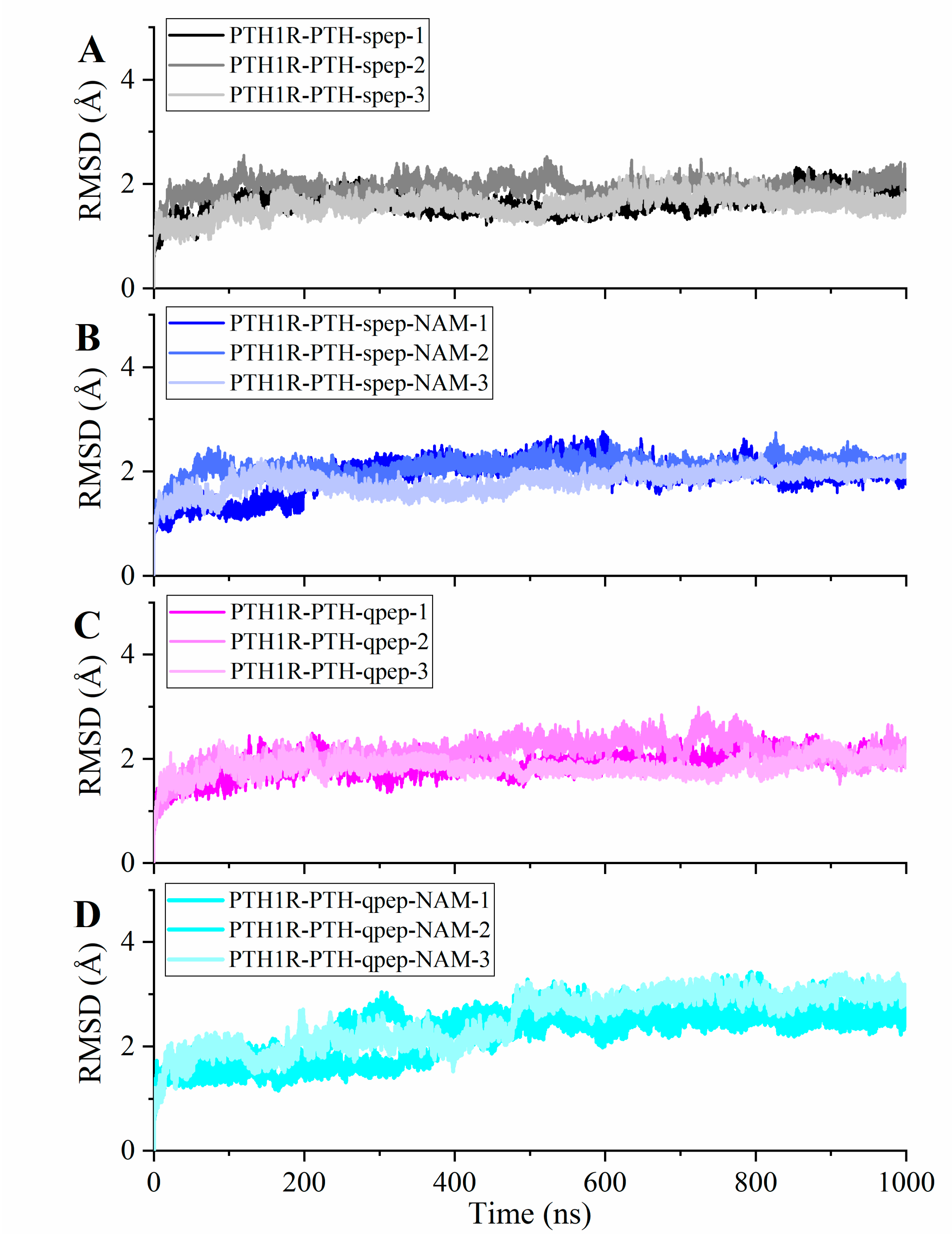

Figure S2. RMSDs of 7TM Cα atoms for all MD simulations. (A) PTH1R-PTH-spep system; (B) PTH1R-PTH-spep-NAM system; (C) PTH1R-PTH-qpep system; (D) PTH1R-PTH-qpep-NAM system.

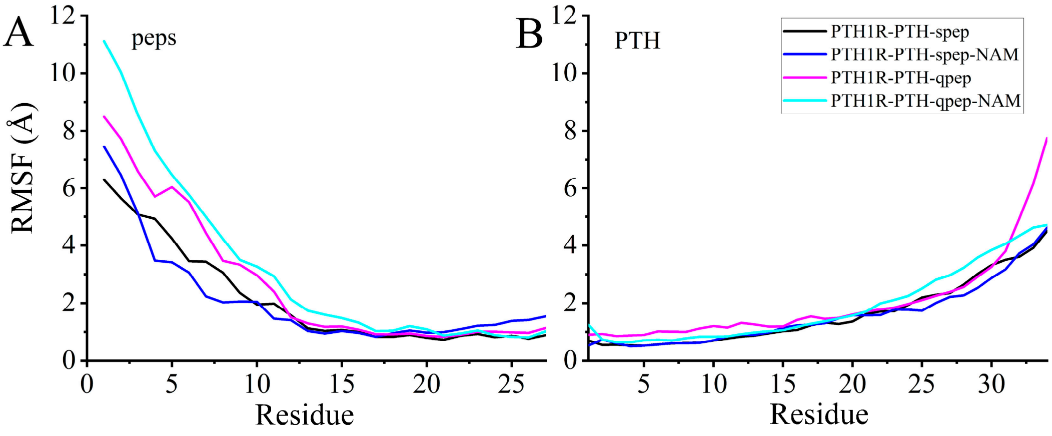

Figure S3. RMSF of peps (A) and PTH (B) in the last 300-ns MD trajectories using their average structures as references.

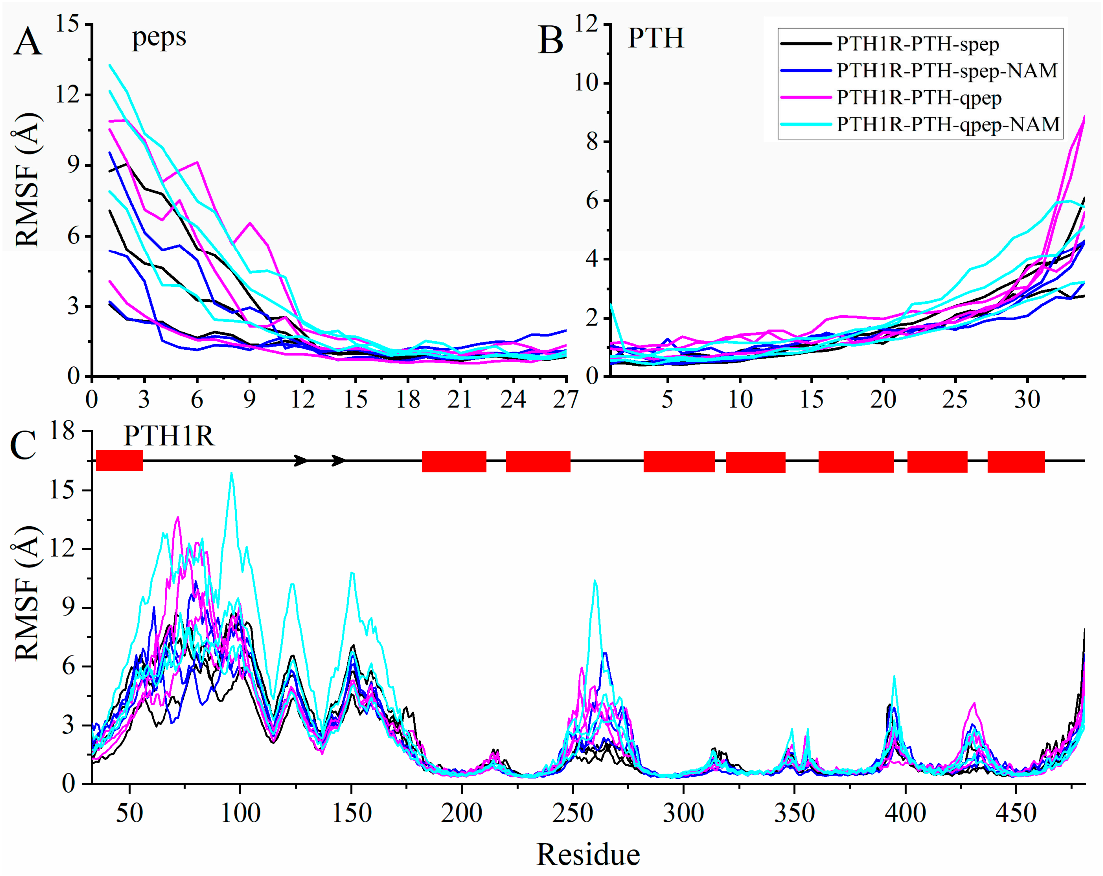

Figure S4. RMSFs of each system in the last 300-ns MD trajectories using average structure as references: (A) peps; (B) PTH; (C) PTH1R. The rectangles and arrows on the top represent ordered secondary structures α-helices and β-sheets, respectively.
